# Sparse Reconstruction for Micro Defect Detection in Acoustic Micro Imaging

**DOI:** 10.3390/s16101773

**Published:** 2016-10-24

**Authors:** Yichun Zhang, Tielin Shi, Lei Su, Xiao Wang, Yuan Hong, Kepeng Chen, Guanglan Liao

**Affiliations:** 1 State Key Laboratory of Digital Manufacturing Equipment and Technology, Huazhong University of Science and Technology, Wuhan 430074, China; zhangyc@hust.edu.cn (Y.Z.); tlshi@hust.edu.cn (T.S.); wangxiao1989@hust.edu.cn (X.W.); hongyuan@hust.edu.cn (Y.H.); ckphust@hust.edu.cn (K.C.); 2 Jiangsu Key Laboratory of Advanced Food Manufacturing Equipment and Technology, Jiangnan University, Wuxi 214122, China

**Keywords:** sparse reconstruction, micro defect, acoustic micro-imaging, point spread function

## Abstract

Acoustic micro imaging has been proven to be sufficiently sensitive for micro defect detection. In this study, we propose a sparse reconstruction method for acoustic micro imaging. A finite element model with a micro defect is developed to emulate the physical scanning. Then we obtain the point spread function, a blur kernel for sparse reconstruction. We reconstruct deblurred images from the oversampled C-scan images based on *l*_1_-norm regularization, which can enhance the signal-to-noise ratio and improve the accuracy of micro defect detection. The method is further verified by experimental data. The results demonstrate that the sparse reconstruction is effective for micro defect detection in acoustic micro imaging.

## 1. Introduction

The most commonly used methods in nondestructive evaluation for micro defect detection are acoustic micro imaging (AMI), X-ray [1,2], and infrared thermography [3]. AMI, also known as scanning acoustic microscopy, has been proven to be sufficiently sensitive for detecting micro defects and features, such as delamination [4,5], voids in the interfaces [6], microbubbles [7,8], stress distributions [9], solder bumps in flip chip [10,11], etc. The general working modes of AMI include A-scan, B-scan, and C-scan, obtaining time-domain signal, time-spatial image, and spatial image, respectively. 

Most investigations have focused on the A-scan signal [12,13,14,15] to improve the resolution of ultrasonic detection by separating the echoes via sparse reconstruction. The sparse reconstruction has also been introduced to improve the resolution of B-scan image [16,17,18]. In these works, the size of the objects was up to a millimeter and the central frequency of the planar immersion transducer or contact transducer was only 5 MHz. Till now, little work has been involved in research on sparse reconstruction for C-scan images of AMI. Actually, C-scan can provide more effective results than A/B-scan for detecting micro defects, and the transducer is a focusing transducer whose central frequency can be as high as 230 MHz [10,11] or even GHz [6]. The acoustic field distribution of the focusing transducer is completely different from the planar transducer, and the formation of the C-scan image is also different from that of the B-scan image. For C-scan mode, the key parameter of sparse reconstruction is the point spread function (PSF), also known as blur kernel in image processing. The PSF describes the response of a transducer system to a point source or reflector placed at the focal plane. By scanning a small target in water, Canumalla obtained the PSF of the transducer with the highest center frequency of 30 MHz [19]. However, the acoustic propagation within the solid specimen is different from the condition that the target is directly immersed in the water. The PSF in the specimen should be different from the one in water. Moreover, the focal zone in the specimen of the high frequency transducer like 230 MHz only has a size of 10s of micrometers, and the target for measuring the PSF should be limited to a few micrometers. Besides, the PSF covered by noise cannot be obtained directly. It is hard to measure the PSF directly for high frequency transducer in AMI. Zhang et al. developed a 2D AMI finite element model of a flip-chip package to investigate the acoustic propagation inside the package [20,21,22,23], while sparse reconstruction and super resolution were not involved.

In this work, we propose a sparse reconstruction method for micro defect detection in AMI. A finite element model which contains a micro defect with unit size is developed. Then we shift the defect to emulate the scanning system, and acquire the PSF of the system. By using the PSF as a blur kernel, we reconstruct the deblurred image from the oversampled C-scan image based on *l*_1_-norm regularization, and improve the accuracy for micro defect detection successfully. The sparse reconstruction was further verified by experimental results, proving its effectiveness in improving resolution and enhancing signal-to-noise ratio for micro defect detection.

## 2. Method

The AMI system working on C-scan is modeled as a linear time invariant (LTI) system,

(1) y=k⊗x+n

where the output signal *y* denotes an observed C-scan image, *k* is a matrix representing the PSF of the AMI system, the input signal *x* (the reflectivity field) denotes an ideal C-scan image, *n* denotes the noise, and ⊗ is convolution operation. When *y* is available and *x* needs to be estimated, the problem is an ill-posed problem where prior image information is required [24]. Considering that the defects in an object are generally finite and their distribution is sparse, only a few pixel values of the image *x* where the defect exists should be non-zero, and the others should be 0. Thus we can add an *l*_1_-norm regularization term to make sure *x* be sparse, and obtain the reconstructed image $\hat{x}$ by solving the optimization problem,

(2) $$\hat{x}=\underset{x}{\arg\min}\;\lambda {\left\|k⊗x-y\right\|}_{2}^{2}+{\left\|x\right\|}_{1}$$

where ${\left\|\;\right\|}_{2}$ denotes the Euclidean norm, ${\left\|\;\right\|}_{1}$ denotes the *l*_1_-norm in vector, and *λ* is the regularization parameter. The first term ensures that the solution is consistent with the observed image, and the second term inserts the image prior information to the solution. The problem in Equation (2) is a typical convex *l*_1_-regularized problem [24,25] that can be solved by iterative shrinkage-thresholding algorithm (ISTA) [26]. The regularization parameter *λ* balances the first term and second term of Equation (2), which means the value is affected by the fidelity of the measurements, the noise level, and the sparse level of the image [26]. For a given C-scan image, a large value will result in a least squares solution which may be unstable, while a small value may over smooth the solution due to excessive regularization [18,26,27,28]. Here, we set *λ* according to the method described in [28].

Then *y* and $\hat{x}$ are evaluated using a target-to-clutter ratio (TCR) metric adapted from [28], which is a measure of the signal in the target region relative to the signal from the background region. The TCR represents the signal-to-noise ratio of the image,

(3) $$TCR=20\log_{10}\left(\left(\frac{1}{N_{T}}\sum_{T}f_{ij})/(\frac{1}{N_{b}}\sum_{b}f_{ij}\right)\right),$$

where $f_{ij}$ denotes the pixel of the image, *T* denotes the target region in the image, *N_T_* is the number of pixels in the target region, *b* denotes the background region, and *N_b_* is the number of pixels in the background region. Due to the *l*_1_-norm regularization, most of the pixel values in the background region will be zero, and the signal-to-noise ratio can be significantly enhanced. We call this method AMI sparse reconstruction (AMISR), whose pseudocode is presented in Algorithm 1. The parameters *δ* and *ε*, both set to 0.001, are the step sizes for ISTA update and convergence tolerance, respectively. The operator *S* is a soft shrinkage operation, defined as $S_{\delta }(x_{i})=max\left\{\left|x_{i}\right|-\delta ,0\right\}\cdot sign(x_{i})$, shrinking every component of the input matrix towards zero.

**Algorithm 1. Pseudocode of AMISR**1: **Input:** Oversampled C-scan image *y*, point spread function *k*, regularization parameters *δ*, *ε*, *λ* 2: **Initialization**
$x_{0}=y$, flag = 1 3: **while** (flag), do 4:  $\upsilon_{k}=x_{k}-\delta \lambda K^{T}(Kx_{k}-y)$ 5:  $x_{k+1}=S_{\delta }(\upsilon_{k})$ 6:  **if**
${\left\|x_{k+1}-x_{k}\right\|}_{2}^{2}<\varepsilon$ 7:   $\hat{x}=x_{k+1}$, flag = 0 8:  **end** 9: **end** 10: Calculate *TCR*11: **Output:** Reconstruct image $\hat{x}$
*, TCR*

## 3. Point Spread Function (PSF)

### 3.1. Modeling

**Figure 1:**
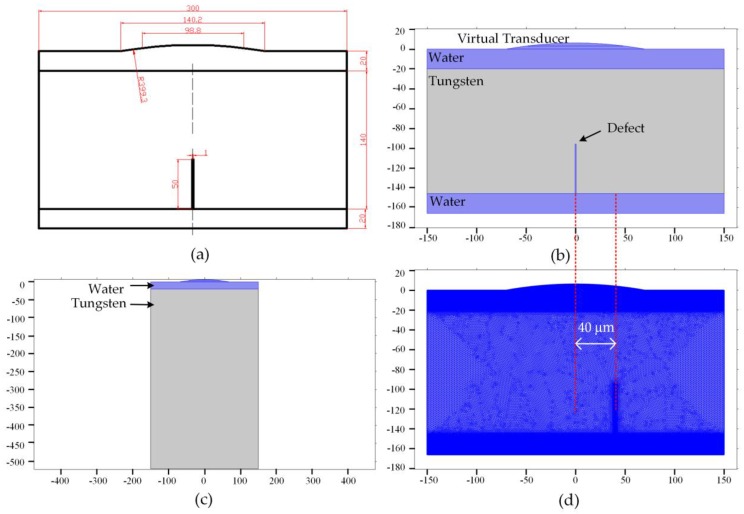
The simulation model (unit in μm ): (**a**) The geometrical model; (**b**) Area segments in the model with defect; (**c**) Area segments in the model without defect; (**d**) Meshing with 10 elements per wavelength, and the position of defect is offset to emulate the transducer scanning in AMI.

The PSF describes the response of the AMI system (with the spatial sampling intervals of 1 μm) to a defect (with the size of 1 μm) placed at the focal plane, i.e., the observed C-scan image. The finite element model is developed with the commercial software (COMSOL Multiphysics 5.2), as shown in Figure 1. To overcome the huge computational resource requirements [20,21,22,23], we design a virtual transducer (VT) by reducing the size of the physical transducer (1/20 of the physical transducer) in the model, as shown in Figure 1a. The sizes of the coupling water zone and the thickness of the sample are also reduced. The arc that represents the VT has a chord length of 98.8 μm and a curvature radius of 399.3 μm. The micro defect has a width of 1 μm and a height of 50 μm, as shown in Figure 1b. The material properties used in the model are obtained from the material library of the COMSOL. The acoustic velocities in water and tungsten are 1484 m/s and 4620 m/s, respectively. The densities of water and tungsten are set to 1000 Kg/m^3^ and 17,800 Kg/m^3^. The Poisson’s ratio and Young’s modulus of tungsten are 0.27 and 360 GPa. The ultrasonic excitation signal is a broadband modulated pulse which can be modeled as a Gabor function,

(3) $$f(t)=A\cdot \exp(-\frac{\pi {\left(t-\mu \right)}^{2}}{\sigma^{2}})\cdot \sin(2\pi f_{0}t)$$

where A is the reference amplitude, *f*_0_ is the central frequency of the transducer (230 MHz), and *μ* = 2/*f*_0_ and *σ* = 1/(2*f*_0_) are the translation and standard deviation of Gaussion function. The Gabor function is defined as the normal displacement of the VT arc during excitation. After the excitation, the VT arc is set as a radiation boundary for receiving signals. The solid-liquid interface is set as an acoustic-structural coupled boundary, and the other boundaries are set as radiation boundaries in the liquid region and low reflection boundaries in the solid region. The grid in the model is set as 10 elements per wavelength. The temporal resolution is set as 25 time steps per period of the acoustic wave. The simulation is carried out for 90 ns, where the excitation lasts for the first 20 ns. The integral of the pressure data along the transducer arc is defined as the received signal of the VT. 

We first carry out transient simulation in tungsten without defect to confirm the position of the focus of the VT, as shown in Figure 1c. Afterwards, the defect is located in the focal plane of the VT with the horizontal position changing at 1 μm intervals in simulations. Since the model is axisymmetric, only half of the model is scanned.

### 3.2. Characteristics of the VT

**Figure 2:**
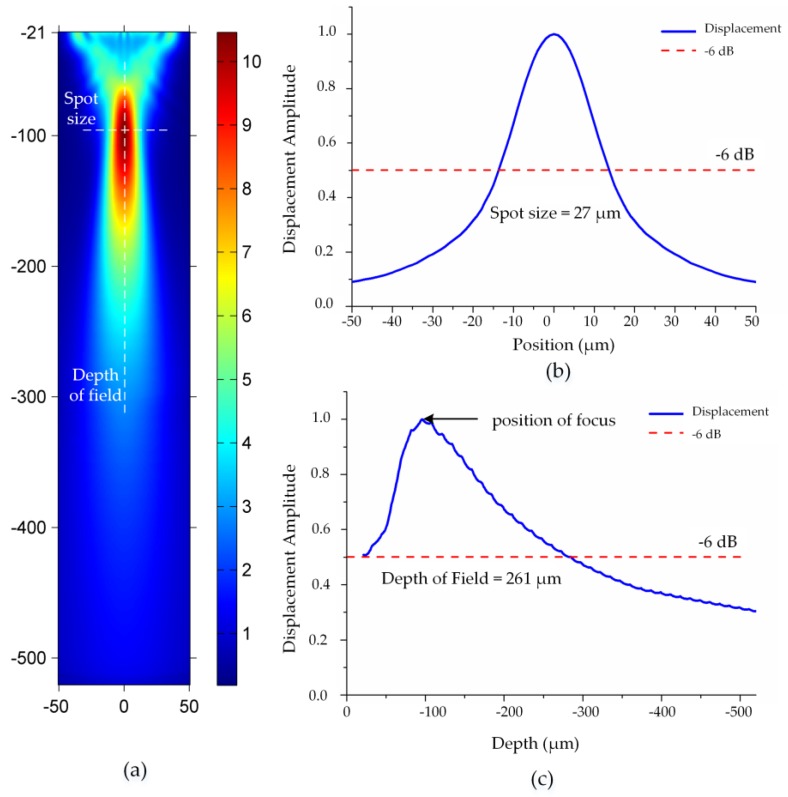
Characteristics of the VT in solid (unit in μm): (**a**) Beam profile of the VT with diagram of DOF and the spot size; (**b**) Spot size measurement in lateral direction; (**c**) Depth of field measurement in vertical direction.

Figure 2a depicts the beam profile obtained from the model (Figure 1c) by mapping maximum displacement of the coordinate points, where an obvious focus region can be observed. Figure 2b,c indicates the normalized displacement along the dash lines labeled in Figure 2a. The red dash lines indicating −6 dB cut-off threshold define the depth of field (DOF) and the spot size of the VT. The spot size in tungsten is measured as 27 μm in Figure 2b. The DOF is given as 261 μm in Figure 2c with the focal plane about −96 μm.

### 3.3. C-Scan and PSF

**Figure 3:**
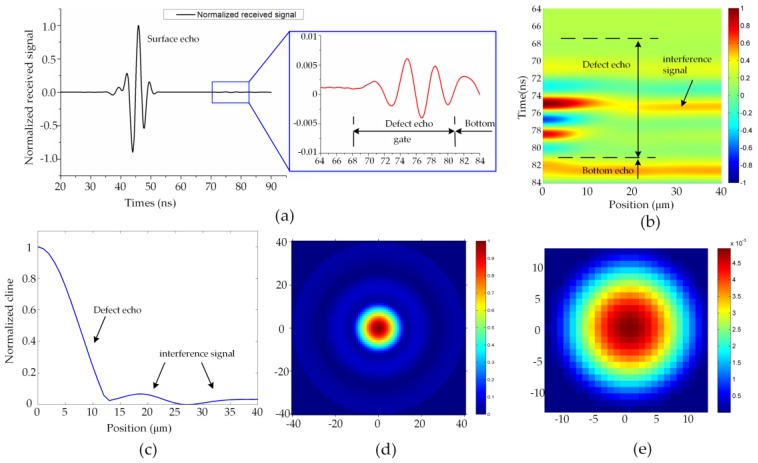
The A-scan, C-scan, and PSF (unit in μm): (**a**) A-scan of the model with the transducer at position 0 μm; (**b**) B-scan like image; (**c**) C-line; (**d**) C-scan; (**e**) PSF extracted from (**d**).

Figure 3a depicts a representative A-scan signal with the transducer at the position 0 μm, which indicates that the transducer is located upon the center of the defect. The echo of the defect is too weak compared with that of the surface, which is zoomed in the insert. The dash lines represent the gate of echo. A-scans are then assembled in Figure 3b like a B-scan image, where only the echo of the defect is shown. Every column of the figure represents an A-scan signal obtained in every simulation with a different position of the defect, and every row represents the varied time of the received signal. The defect echo gated by the dash lines is the received signal related to the defect. Some interference signals are observed when the transducer is far away from the defect, which may be caused by the sidelobe of the focus region as shown in Figure 2a.

The maximum amplitude values of the gated A-scan signals are calculated, forming the C-line as displayed in Figure 3c. Obviously, it decreases when the transducer moves away from the defect. The C-scan is produced after mapping the C-line around the central axis, as shown in Figure 3d. By abandoning the small values caused by the interference signal, we acquire the PSF and normalize it as illustrated in Figure 3e, which can be used for further analysis. 

## 4. Experimental Verification

**Figure 4:**
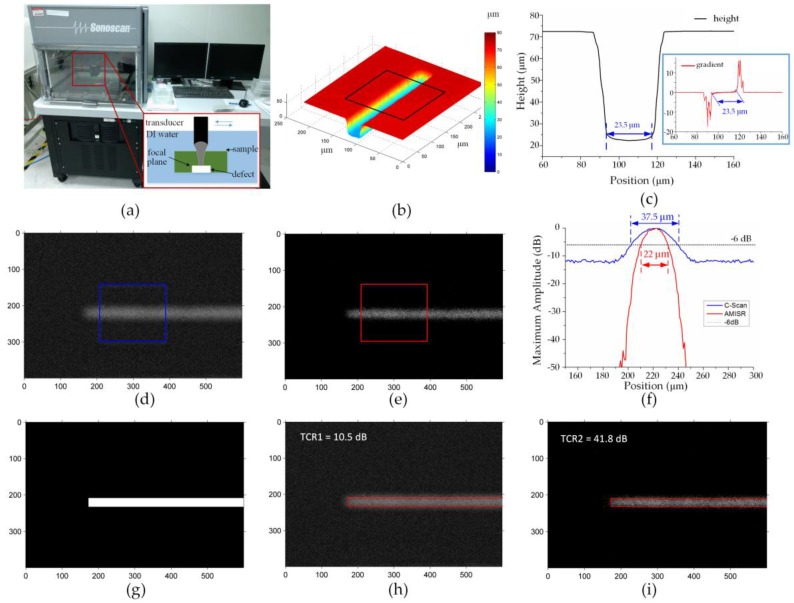
Results of the single groove (unit in μm): (**a**) Schematic diagram of AMI; (**b**) The topography of the groove; (**c**) Average profile of the groove; (**d**) Original C-scan image; (**e**) The reconstructed image formed by AMISR; (**f**) Average values of the regions indicated in (d) and (e); (**g**) The cross section of the groove; (**h**) The original C-scan image superimposed with the mask; (**i**) The reconstructed image superimposed with the mask.

Three samples with artificial defects (including single groove, two grooves, and a complex defect) were used for verification. A circular tungsten sheet 4 inches in diameter and 500 μm in thickness was polished to optical flatness on both sides. Then the defect pattern was etched with the depth about 50 μm by inductive couple plasmas (ICP) etching. After that, the sheet was cut into small pieces with the area about ~10 × 10 mm^2^. The AMI test was performed on the pieces using a scanning acoustic microscopy (SAM, D9500, Sonoscan, Elk Grove Village, IL, USA), as shown in Figure 4a. The transducer (230SP) had a central frequency of 230 MHz and a focal length of 7924.8 μm in water. The f-number of the transducer was 4. The pieces were immersed in water and the focus of the transducer was located at the bottom of the artificial defects. The spatial sampling interval of the scanning system was 1 μm for 1 pixel, in accord with the simulation. Finally, the topography of the artificial defects was measured with a laser scanning confocal microscopy (LSCM, VK-X200K, Keyence, Japan) for comparison.

### 4.1. Single Groove

Figure 4b displays the topography of the single groove obtained by LSCM, and the average height of the region indicated by the black box is given in Figure 4c. The sidewall of the groove has a slight tilt caused inevitably by the ICP process. To determine the bottom and the sidewall, first-order derivative of the height curve is shown in the insert. The region where the derivative almost equals zero is considered as the bottom, of which the width is 23.5 μm. 

The original C-scan image and reconstructed image by the AMISR are presented in Figure 4d and Figure 4e, respectively. To avoid the interference of the noise, the average profiles along the direction of the groove are presented in dB (Figure 4f). The blue line and red line represent the average profiles calculated from the blue block in Figure 4d and the red block in Figure 4e. Here the −6 dB width is defined as the width of the groove. We obtain the width of 37.5 μm (according to the original C-scan image) and 22 μm (according to the reconstructed image). Compared with the width obtained from the LSCM, the AMISR provides a more accurate result (only 6.4% deviation) than the original C-scan (59.6% deviation).

To quantify the image formed by the AMISR and C-scan, TCR is calculated to analyze the signal-to-noise ratio. The cross section of the groove based on the reference values is generated as a mask (illustrated in Figure 4g). To distinguish the target region from the background region, the mask is superimposed on the original C-scan image (Figure 4h) and the image formed by AMISR (Figure 4i). The TCR of the AMISR result is 41.8 dB, almost four times that of the original C-scan (10.5 dB). Hence, the proposed method can significantly enhance the signal-to-noise ratio.

### 4.2. Two Grooves

**Figure 5:**
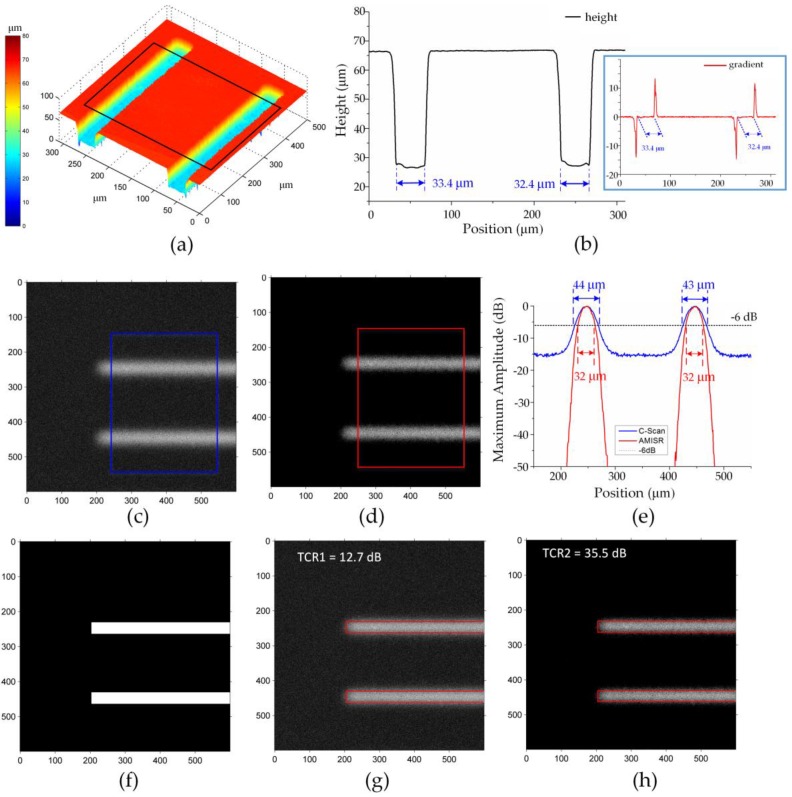
Results of two grooves (unit in μm): (**a**) The topography of the grooves; (**b**) Average profile of the grooves; (**c**) Original C-scan image; (**d**) The reconstructed image formed by AMISR; (**e**) Average values of the regions indicated in (c) and (d); (**f**) The cross section of the grooves; (**g**) The original C-scan image superimposed with the mask; (**h**) The reconstructed image superimposed with the mask.

The topography of the two grooves obtained by the LSCM is shown in Figure 5a. The average profile indicated by the black box in Figure 5a illustrates the reference widths of the two grooves (33.4 μm and 32.4 μm), as shown in Figure 5b. The original C-scan image, presented in Figure 5c, gives the width of 44 μm or 43 μm as shown in Figure 5e, 31.7% or 32.7% deviated from the reference values. The reconstructed image formed by the AMISR, as shown Figure 5d, provides the widths about 32 μm, only 4.2% or 1.2% deviated from the reference values. Figure 5f–h illustrate the cross sections of the grooves generated on the reference values as a mask, the original C-scan image, and the reconstructed image superimposed with the mask, respectively. The TCR value of the AMISR result is 35.5 dB, almost three times that of the original C-scan image (12.7 dB). Therefore, the proposed method works better than the original C-scan image.

### 4.3. Complex Defect

**Figure 6:**
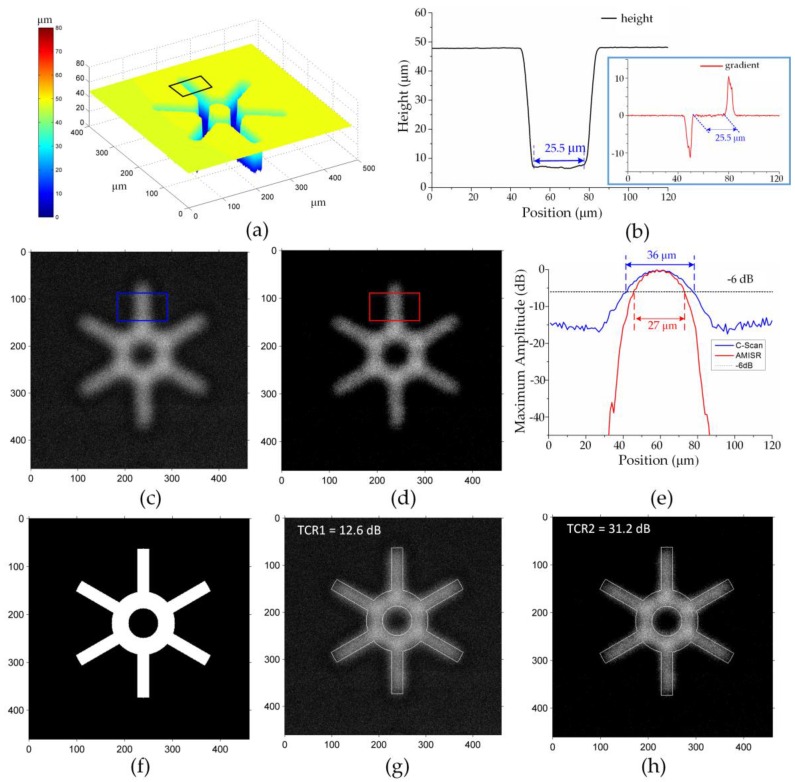
Results of complex defect (unit in μm): (**a**) The topography of the defect; (**b**) Average profile of the branch; (**c**) Original C-scan image; (**d**) The reconstructed image formed by AMISR; (**e**) Average profile of the regions indicated in (c) and (d); (**f**) The cross section of the defect; (**g**) The original C-scan image superimposed with the mask; (**h**) The reconstructed image superimposed with the mask.

Experimental verification on a complex defect is further performed, as shown in Figure 6. The reference width of the branch is 25.5 μm obtained from the LSCM, as shown in Figure 6a,b. The original C-scan image (Figure 6c) gives the width of 36 μm in Figure 6e, 41.2% deviated from the reference value. The reconstructed image (Figure 6d) gives the width of 27 μm with 5.9% deviation. The TCR value of the image formed by the AMISR is 31.2 dB (Figure 6h), better than that of the original C-scan image (12.6 dB in Figure 6g). Thus, the proposed method is also effective for complex defects in resolution improvement and signal-to-noise ratio enhancement.

Here we only focus on the C-scan image and the lateral resolution. The axial resolution can be acquired from the B-scan image, of which the image-forming method is different and also has a corresponding PSF. If we obtain the corresponding PSF, the AMISR can also improve the axial resolution.

## 5. Conclusions

In this paper, a sparse reconstruction method for micro defect detection in AMI is proposed. The AMI system is modeled as an LTI system, where the output signal is the observed C-scan image and the input signal is the ideal C-scan image. When the observed C-scan image is available, the problem of estimating the ideal C-scan image is a deblurring problem, where the key parameter is the PSF. Here, we develop a finite element model for AMI to simulate the detection of micro defects and acquire the PSF of the system. By using the PSF as the blur-kernel, we reconstruct the deblurred image from the oversampled C-scan image based on *l*_1_-norm regularization, improving the resolution and enhancing signal-to-noise ratio. Single groove and two grooves together with a complex defect are used for verification. The experimental results prove that the method has a significant improvement in detection accuracy and signal-to-noise ratio compared to the original C-scan. 

## Abbreviations

The following abbreviations are used in this manuscript:

AMI	Acoustic microscopy imaging
SAM	Scanning acoustic microscopy
PSF	Point spread function
TCR	Target-to-clutter ratio
LTI	Linear time invariant
ISTA	Iterative shrinkage-thresholding algorithm
AMISR	Acoustic microscopy imaging sparse reconstruction
VT	Virtual transducer
ICP	Inductive couple plasmas
LSCM	Laser scanning confocal microscopy
